# Is Your Patient Hungry? Screening for Food Insecurity in Dental Education

**DOI:** 10.1002/jdd.70057

**Published:** 2025-09-22

**Authors:** Teresa A. Marshall, Nadine Tassabehjim, Khatija Noorullah, Christie Custodio‐Lumsden, Jillian Kaye, Rena Zelig, Riva Touger‐Decker

**Affiliations:** ^1^ Department of Preventive and Community Dentistry, College of Dentistry University of Iowa Iowa City Iowa USA; ^2^ Department of Comprehensive Care Tufts University School of Dental Medicine Boston Massachusetts USA; ^3^ Division of Preventive and Public Health Sciences, College of Dentistry University of Illinois Chicago Chicago Illinois USA; ^4^ Section of Oral, Diagnostic, and Rehabilitation Sciences Division of Foundational Sciences Columbia University College of Dental Medicine New York City New York USA; ^5^ Department of Pediatric Dentistry New York University College of Dentistry New York City New York USA; ^6^ Department of Clinical and Preventive Nutrition Sciences Rutgers School of Health Professions Rutgers, The State University of New Jersey Newark New Jersey USA; ^7^ Rutgers School of Health Professions Rutgers, The State University of New Jersey Newark New Jersey USA

## 1 Introduction

A healthy oral cavity enables essential functions such as eating, drinking, and speaking, allowing individuals to live without pain, discomfort, or embarrassment [1]. As such, oral health encompasses much more than the absence of disease. Achieving and maintaining oral health throughout the lifespan depends on collaboration between the oral health care provider (OHCP) and the individual. OHCPs are expected to provide anticipatory preventive oral health guidance while treating existing disease, and individuals are expected to perform routine oral hygiene and adopt healthy lifestyle practices while minimizing exposures that increase the risk of oral and systemic disease. As part of preventive guidance, OHCPs often recommend that patients increase intakes of fruits, vegetables, and dairy foods while lowering intakes of added sugars by reducing consumption of sugar‐sweetened beverages, candy, and other processed foods and beverages both between and with meals. Such recommendations assume the patient has a choice; that is, the patient has the resources and access to procure healthier food items in their lived environment. Are such assumptions relative to food choice valid? How do we know?

## 2 Main Section

Food security is defined as having access to sufficient, safe, and nutritious foods to meet daily needs [2]. Food insecurity (FI) is a social determinant of health (SDH) defined as having limited or uncertain access to adequate food to meet daily needs. In 2023, the prevalence of any FI, low food security, and very low food security was 13.5%, 8.4%, and 5.1%, respectively, in the United States [2]. FI is higher for households with versus without children, headed by single mothers, composed of racial minorities, and/or living in poverty [2].

Individuals living with FI often have limited availability of transportation to full‐service grocery stores and inadequate financial resources to access healthy foods, resulting in higher intakes of inexpensive ultra‐processed foods. Although readily accessible and convenient, ultra‐processed foods, including hot dogs and cold cuts, sugar‐sweetened beverages, cereals, snack foods, and desserts, are typically of high energy density, low nutrient density, and high in added sugars. Higher diet quality is associated with higher food cost. As a result, individuals from lower socioeconomic status groups, including those with FI, typically have lower‐quality diets than their peers from higher socioeconomic status groups. FI is associated with an increased risk of diet‐related, non‐infectious systemic diseases, including obesity, type 2 diabetes, cardiometabolic diseases, and cancers.

Dental caries is a diet‐dependent disease; without fermentable carbohydrates, caries do not occur. Although periodontal disease is not directly caused by diet‐ or nutrient‐related factors, adequate nutrient intakes are necessary to support soft tissue health and immune function. Cardiometabolic disease, associated with a higher risk of periodontal disease, is also higher in individuals consuming ultra‐processed foods with low nutrient intakes. Oropharyngeal cancers are associated with lower intakes of fruits and vegetables and higher intakes of processed meats. Thus, a healthy diet is necessary to support oral health. The relationship between oral health and a healthy diet is bidirectional, as oral health also facilitates the consumption of a healthy diet. An increased prevalence of caries, periodontal disease, and oropharyngeal cancers is associated with lower Healthy Eating Index scores, a measure of overall diet quality.

Likewise, FI is associated with a greater prevalence of caries and periodontal disease. Drumond et al. conducted a meta‐analysis and reported that individuals with FI were more likely to have caries than individuals with food security (OR = 1.66; 95% CI: 1.36–2.02) [3]. FI has been associated with periodontal disease using NHANES data; the predicted probability of periodontal needs increased with increasing severity of FI [4]. Thus, FI is not only a barrier to a healthy diet but also a barrier to oral and systemic health. To provide effective dietary counseling that enables a patient to reduce their risks of diet‐related caries, periodontal disease, and oropharyngeal cancers and improve overall diet quality, the OHCP must individualize dietary recommendations to the patient consistent with their food security status. Food security status is readily identified through screening protocols. Ideally, early interventions addressing FI facilitate preventive care and lower the likelihood of costly interventions in the future.

As educational institutions, the goal of dental schools is to prepare students to provide comprehensive care to maintain and improve the oral health status of individuals and populations. Faculty guide students to screen, diagnose, and treat patients with oral diseases as well as provide recommendations to promote oral health and prevent oral infectious diseases. As part of comprehensive care, some dental schools are also screening for chronic diseases such as hypertension, diabetes, and cardiovascular disease. In addition to preparing students to screen for and address disease, dental schools have an obligation to prepare students to identify and address SDH, which can increase disease risk and preclude patient participation in their care plan [5]. Patients who seek oral health care at dental schools are typically disadvantaged, have low socioeconomic status, and present with financial and transportation barriers. The demographic profile of many patients seeking care at dental schools is consistent with being at risk of FI. Mays et al. reported that 34% of respondents to a survey designed to identify unmet SDH needs within the University Of Minnesota School Of Dentistry experienced FI [5].

Dental schools teach students how to screen for diet‐related caries and periodontal disease risks and provide appropriate dietary recommendations. Ideally, the identification of achievable dietary recommendations is a negotiation with the patient. There are multiple avenues to achieving a healthy diet based on food group adequacy and meal structure. Fundamental to compliance with the negotiated recommendations is having access to appropriate foods and beverages. If FI is a barrier to compliance with dietary guidance, then the FI precludes the patient's ability to improve their oral and systemic health.

In addition to didactic SDH instruction that presents FI definitions, FI epidemiology, and associations between FI and both oral and systemic disease, dental education should prepare students to screen for and address SDH, including FI, in their patients. Faculty modeling FI awareness, FI screening, and addressing FI in the patient population and student experience will increase student comfort and encourage continued behavior in their future practice. Screening for FI improves clinician awareness of SDH, builds trust with their patients, and advances holistic patient‐centered care.

To facilitate patient screening for FI, dental schools are encouraged to include screening instruments in their electronic health records. The ‘*Hunger Vital Sign’* is a two‐item FI screen initially validated in a pediatric clinic, supported by the Children's Health Watch, and currently used in a limited number of medical and dental schools [6, 7]. Numerous health professional organizations, including the American Academy of Pediatrics, the Centers for Medicare & Medicaid Services, and the Academy of Nutrition and Dietetics, support screening for FI in healthcare settings [7, 8]. Although clinicians support FI screening, barriers to implementing screening protocols include clinician discomfort, time commitment, concerns related to alienating patients, addressing positive responses, and limited knowledge of community resources [9]. These barriers can be addressed through faculty and clinician education, collaboration with other health professionals (i.e., social workers, registered dietitian nutritionists), and experience. Inclusion of the *Hunger Vital Sign* screen in electronic health records as part of medical histories or other intake forms can normalize the screening process, provide experience for dental students, and support interprofessional collaboration.

The Hunger Vital Sign is based on the US Household Food Security Module [6]. Respondents are asked if either or both of the following two statements are often true, sometimes true, or never true:

1. *Within the past 12 months, we worried whether our food would run out before we got money to buy more*.
2. *Within the past 12 months, the food we bought just didn't last, and we didn't have the money to get more*.

An often true or sometimes true response to one or both statements is consistent with FI.

Prior to screening for FI in the clinic setting, administrators must identify actions to assist respondents who screen at risk for FI and communicate these actions to both faculty and students. Depending on the nature of the dental school, social workers or registered dietitian nutritionists might lead the process of identifying resources to address FI or connecting patients with community health resources. Actions to assist respondents with FI might include patient education tools providing a list of phone numbers for the Supplemental Nutrition Assistance (SNAP) and Women, Infants, and Children (WIC) programs, as well as local food banks, food pantries, and soup kitchens. The patient education tools can be made available online for students and faculty to print on demand to give to patients at risk for or with FI. Kopparapu et al. found that over 70% of FI adults surveyed at academic university or neighborhood clinics responded that the following information would be helpful: Referral for financial assistance programs for which one qualified, a list of food bank locations, a list of farmer market locations, and referral to local community organizations [10]. Consistent with principles of patient‐centered care, administrators and faculty should emphasize the importance of employing empathy and compassion when engaging patients in FI screening and facilitating connections to FI resources.

FI screening is part of the information‐gathering process; provision of resource information is most relevant when FI is identified. Resources can also be provided during treatment planning or reintroduced during the dietary counseling phase. Knowledge of a patient's food security status enables patient‐centered dietary guidance to support healthier eating. Acknowledgement of barriers is appropriate when negotiating strategies to improve diet quality to improve both oral and systemic health. Presentation of resource information to alleviate barriers, consultation with other health care providers, and referral for community resources may be necessary to support patients in achieving healthier dietary behaviors. Furthermore, OHCPs can advocate for food security in their local communities to address health disparities and promote public health.

## 3 Summary

Becoming a competent OHCP requires students to acknowledge individual SDH that limit patient participation in their oral healthcare and identify strategies to address the SDH barrier. FI is prevalent in the patient population that seeks care in dental schools and is a barrier to oral and systemic health. Preparing students to screen for and address FI starts with faculty leadership. Faculty must model practices to ensure that FI screening is integrated into the dental clinic setting. Providing students with both the tools to identify FI and resources to help patients access healthy foods is a critical step toward empowering patients to make informed food choices. This, in turn, supports patients’ active participation in their oral health care and contributes to better oral and systemic health outcomes.

## Conflicts of Interest

The authors declare no conflicts of interest.
